# Obstructive sleep apnea syndrome (OSAS) and hypertension: Pathogenic mechanisms and possible therapeutic approaches

**DOI:** 10.3109/03009734.2012.707253

**Published:** 2012-10-30

**Authors:** Wang Zhang, Liang-yi si

**Affiliations:** Department of Geriatrics, the First Affiliated Hospital, Third Military Medical University, Chongqing, China

**Keywords:** Approach, hypertension, obstructive sleep apnea syndrome, pathogenic mechanism

## Abstract

Obstructive sleep apnea syndrome (OSAS), a chronic condition characterized by collapse of the pharynx during sleep, has been increasingly recognized as a health issue of growing importance over the last decade. Recently emerging evidence suggests that there is a causal link between OSAS and hypertension, and hypertension represents an independent risk factor in OSAS patients. However, the pathophysiological basis for patients with OSAS having an increased risk for hypertension remains to be elucidated. The main acute physiological outcomes of OSAS are intermittent hypoxia, intrapleural pressure changes, and arousal from sleep, which might induce endothelial dysfunction, sympathetic activation, renin–angiotensin–aldosterone system activation, lipid metabolism dysfunction, and increased oxidative stress. This brief review focuses on the current understanding of the complex association between OSAS and hypertension.

## Introduction

Sleep-related breathing disorders lead to concomitant alterations in the central nervous and cardiovascular systems, and such alterations often induce additional health issues. Sleep-disordered breathing represents a wide spectrum of sleep-related breathing abnormalities. Obstructive sleep apnea (OSA) is a condition in which there is repetitive partial or complete collapse of the pharynx during sleep. OSA associated with excessive daytime sleepiness is commonly called obstructive sleep apnea syndrome (OSAS). OSAS is the most common sleep-disordered breathing abnormality (1,2) and often results in apnea or hypopnea, which can lead to snoring.

Our understanding of OSAS has evolved from initially regarding it as merely an annoying social situation to the recognition that OSAS may act through various mechanisms to increase cardiovascular risk and lead to increased morbidity and mortality (3). In particular, there is evidence that untreated OSAS may contribute to the pathophysiological mechanisms underlying the origin and/or development of hypertension, cardiac ischemia, myocardial infarction, congestive heart failure, and stroke (4). Of the different possible consequences of OSAS in patients, the most widely recognized may be the development of systemic hypertension. While many reviews have described the association between OSAS and hypertension, the diagnosis, prevalence, etiology, and new mechanisms linking OSAS to hypertension are outlined in this review.

## Definition and diagnosis of OSAS

OSAS symptoms, including habitual and intermittent snoring, recurrent arousal during sleep, excessive daytime sleepiness, and witnessed apneas suggest the occurrence of OSAS. However, the gold standard diagnostic test for OSAS is the overnight in-laboratory polysomnography. Polysomnography uses multi-channel continuous recordings for electrocardiography, electromyography, electroencephalography, electro-oculography, nasal airflow, snoring sounds, blood oxygen saturation, thoracic and abdominal impedance belts for respiratory effort, and intra-esophageal pressure. The apnea-hypopnea index (AHI), defined as the average number of apneas and hypopneas per sleep hour, has always been used to evaluate OSAS severity. An apnea is defined as the complete airflow cessation for at least 10 seconds (5). Hypopnea may happen during sleep or awake states and fluctuate in severity of episodes of shallow breathing or an abnormally low respiratory rate. According to the American Academy of Sleep Medicine Task Force recommendations, OSAS is defined as an apnea-hypopnea index (AHI) >5, along with excessive daytime somnolence (6). Another diagnosis standard of OSAS is the number of apneas (where airflow stops completely) and obstructive hypopneas (> 50% reduction in respiratory flow or > 30% reduction linked to more than 3% desaturation and/or microarousals) lasting more than 10 seconds per hour. A threshold of 15 events per hour of recording is applied for OSAS diagnosis (7). OSAS is further subclassified into mild OSAS (AHI = 5–15), moderate OSAS (AHI = 16–30), and severe OSAS (AHI >30). A habitual snorer is a subject who always snores at night, while the AHI is <5 (6).

## Epidemiology of OSAS

The prevalence of OSAS varies among study populations, due to the use of different variables, and is influenced by the criteria used to define OSAS as well as by the population characteristics. The first large epidemiologic polysomnographic study of OSAS was done in 1995 (8). Later, many studies reported the adult prevalence of OSAS in many different countries and among different ethnic groups (9–15). The overall estimated prevalence of OSAS is in the range of 3%–7% in adult men and 2%–5% in adult women (16), with certain subgroups of the population bearing higher risk. These subpopulations include overweight or obese people and middle-aged and older subjects. Notably, the results that there was no substantial difference in OSAS prevalence in North America, Europe, Australia, and Asia clearly suggest that OSAS is common not only in developed countries, but also in developing countries. In 2008, the American Heart Association and the American College of Cardiology distributed a joint scientific statement pointing out that 85% or more of humans with clinically significant OSAS have not been diagnosed (17). The referral populations of OSAS patients may represent only the tip of the iceberg of OSAS prevalence.

## Evidence connecting hypertension to OSAS

OSAS is widespread in the middle-aged and older population, while hypertension is also highly prevalent among the middle-aged and older population. This raises the possibility of considerable co-morbidity between hypertension and OSAS. A lot of experimental and clinical evidence has indicated that the extent of the co-morbidity of hypertension and OSAS is actually substantially greater than expected.

In animal studies, direct evidence of the relationship between OSAS and hypertension has been well-established. Experimental OSAS resulted in acute transient increases in nighttime blood pressure (BP) and eventually produced sustained daytime hypertension (18). Acute normalization of blood pressure reduced sleep apneas in rats, even in the context of lifelong hypertension (19). Troncoso Brindeiro et al. developed a model of sleep apnea by exposing rats to chronic intermittent hypoxia (CIH) during sleep and found that this protocol increased blood pressure (20).

In humans, a large number of studies have sought to determine the presence and extent of a causal relationship between OSAS and hypertension, independent of frequently co-occurring and possibly confounding variables including age, body weight, and body mass index. The most convincing evidence for the support of a causal relationship between OSAS and hypertension has come from numerous epidemiological data collected in community populations. Early studies indicated that hypertension was found in about 50% of OSAS patients (21), while about 30% of hypertensive patients also have OSAS (22–25). The Wisconsin Sleep Cohort Study analyzed data on sleep-disordered breathing, blood pressure, habitus, and health history, at baseline and after 4 years of follow-up, in 709 Wisconsin state employees, using attended full polysomnography (26–28). Relative to the reference category of an apnea-hypopnea index of 0 events per hour at baseline, the odds ratios for the presence of hypertension was 2.03 (95% confidence intervals 1.29–3.17) in subjects with an apnea-hypopnea index of 5.0 to 14.9 events per hour, suggesting that the presence of hypertension was independent of known confounding factors and that sleep-disordered breathing is likely to be a risk factor for hypertension and subsequent cardiovascular morbidity in the general population (26). Moreover, the non-dippers exhibited a blunting of the sleep-related fall in blood pressure and an increased variability in blood pressure associated with sleep-disordered breathing (29). These results have been partly or completely confirmed by additional independent studies (30–35).

Some researchers have concluded that an increase in diastolic blood pressure might be the earliest hypertensive change associated with OSAS, evidenced by the fact that diastolic blood pressure was higher early in the course of OSAS (36), and diastolic and systolic-diastolic hypertension were the prominent types of hypertension observed both by clinical and ambulatory measurements (37). On the contrary, Sin et al. demonstrated that the systolic blood pressure was significantly higher in patients with OSA than in patients without OSA, indicating a high prevalence of systolic hypertension in patients with OSA (38). In addition, systemic hypertension was associated with a greater exacerbation of blood pressure variability in OSAS patients during sleep (39). The baroreflex sensitivity, an index of the cardiovascular control, was lower during wakefulness and rapid eye movement sleep in untreated OSAS patients than in normal subjects, and negatively correlated with the increase of blood pressure after apneas (40). Therefore, it seems that hypertension, increased blood pressure variability, and decreased baroreflex sensitivity may tightly correlate with OSAS and contribute to the increased cardiovascular risk of OSAS.

## The similar risk factors in OSAS and hypertension

**Figure 1.**
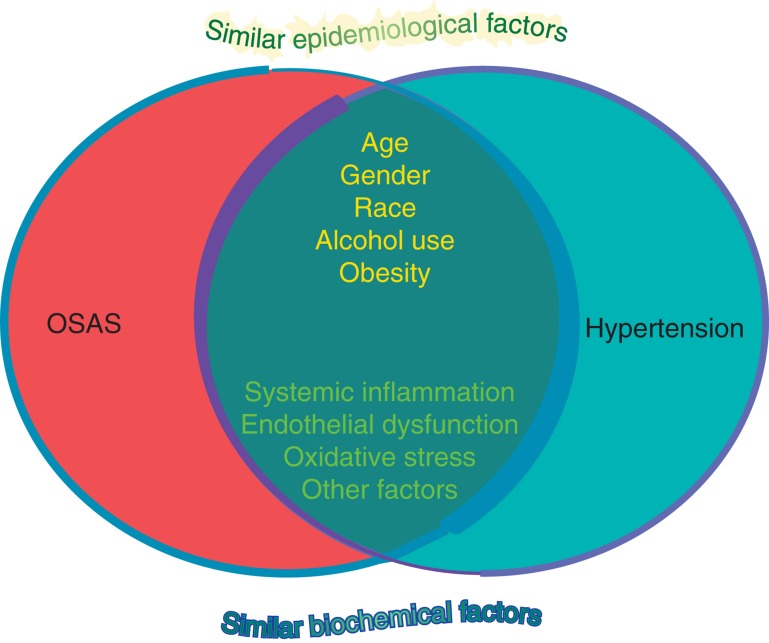
Convergence of epidemiological and biochemical variables in patients with OSAS and essential hypertension.

The pathophysiological pathways linking risk factors between OSAS and hypertension intersect with upper airway dilator muscle activity abnormalities, reduced arousal from sleep, reduced lung volume, and impaired ventilatory control stability (41). Community-based studies have identified some major risk factors for OSAS, including age, gender, and obesity. In parallel, these factors are also risk factors of hypertension (1,5). We summarize the similar risk factors between OSAS and essential hypertension in Figure 1, and discuss them below.

### Age

The prevalence of OSAS increases with age (42-44). In older persons (≥ 65 years), the prevalence of OSAS is 2- to 3-fold higher than that in middle-aged individuals. However, the clinical and prognostic impact of OSAS in elderly patients appears lower than in young and middle-aged patients (11,41,45). Several studies have been conducted to identify the cause of the relationship between age and OSAS, but it seems that no consensus has been reached. Eikermann et al. reported that increasing age was correlated with both pharyngeal collapsibility and increase in pharyngeal resistance independent of body mass index (BMI) and gender during sleep, suggesting that the increased prevalence of OSAS in elderly individuals may mainly involve the dysfunction of muscles surrounding the parapharyngeal area (46). The prevalence of hypertension also increases with age (47). However, there is no satisfactory answer to the concurrent impact of age on OSAS and hypertension.

### Obesity

During the past three or four decades, the United States has witnessed a dramatic increase in the number of overweight or obese individuals, which has become a public health issue (48,49). Epidemiologic studies from Europe and North America have clearly identified body weight as the strongest risk factor for OSAS. Respiratory function was certainly affected by obesity, and obesity could induce the collapse of upper airways during sleep (50). In addition, the frequency of respiratory events during sleep rises when body weight increases. An early report indicated that excess body weight was present in >60% of the sleep apnea patients (51). In the Wisconsin Cohort Study, a 10% weight gain predicted an approximately 32% increase in AHI and was associated with a 6-fold increased risk of OSAS, whereas a 10% weight loss predicted a 26% decrease in AHI (44), suggesting that the incidence of OSAS increases along with the increased incidence of obesity.

Longitudinal data from the Cleveland Family Study demonstrated that AHI was significantly associated with BMI and waist–hip ratio (52). Recently, it was demonstrated that among Americans aged 30–69 years, approximately 40% of adults with sleep-disordered breathing had BMIs ≥ 25 (53), indicating obesity being a major risk factor for development of OSAS. Obesity is associated with anatomic changes that predispose to obstruction of the upper airway during sleep. These alterations may generate excess adiposity around the pharynx. Thus, the upper airway could be narrowed by the increases of adipose tissue in the neck and around the upper airway (54,55). Moreover, central obesity is associated with decrease of lung volume (56), which may cause an increase in pharyngeal collapsibility due to a loss of caudal traction of the upper airway (57–59).

However, in the Asian population, where visceral obesity is less prevalent, the prevalence of OSAS is not proportionately reduced. This phenomenon suggests that there may be a craniofacial effect interacting with body habitus (60). With obesity, the extra bulk of adipose tissue around the neck narrows the airway. A narrowed airway would increase the chances of airway collapse and closure during sleep. Mortimore and colleagues demonstrated that truncal and upper body obesity may be superior predictors of OSAS compared to body mass index, because of more fat deposition in the upper airway or pharynx (61). Flemons et al. also showed that increased neck circumference, which may be a marker for localized obesity, increased the risk of OSAS (62). Several imaging studies of patients with OSAS showed larger lateral parapharyngeal adipose tissues and pharyngeal walls in the neck and upper airway compared with non-obese controls (63). However, there have also been some inconsistent reports. Using magnetic resonance imaging, Schafer et al. reported that AHI significantly correlated to the amount of intra-abdominal fat, whereas there was no association between AHI and the size of parapharyngeal fat pads or subcutaneous fat of the neck region (64). The discrepancies may arise from different sets of populations and need to be further investigated.

This increasing prevalence of obesity and obesity-related hypertension has not only been described in the Western developed countries but also in developing countries such as China and India (65). Although obesity undoubtedly is a major risk factor of hypertension and OSAS, respectively, there is a lack of solid and clear clinical and experimental evidence for the in-depth mechanisms underlying the involvement of obesity between hypertension and OSAS. From a clinical viewpoint, nevertheless, a pragmatic approach to treating OSAS and preventing hypertension-related events would be desirable by treating obesity or reducing body weight.

### Ethnicity

Most of the clinical population-based studies on OSAS prevalence were conducted in USA, Europe, and Australia. Recently, in Asian countries, including China, India, and Korea, several studies have been undertaken to characterize the burden of OSAS (13,14,34,66–75). The prevalence of OSAS in Asians is comparable to that documented in published reports of European and American populations. On the contrary, some studies suggest that the situation of OSAS may differ by race.

The overall prevalence of sleep-disordered breathing was approximately equal in Caucasians (30%) and African-Americans (32%), but the severity was higher in the African-American population (76). The odds ratio for severe sleep-disordered breathing was 2.55 for African-Americans compared to Caucasians, even after adjustment for BMI, sex, and age. In the Cleveland Family Study, Redline et al. found that African-Americans with sleep-disordered breathing were younger than Caucasians with sleep-disordered breathing. In addition, they showed that the association of body mass index with OSAS was stronger in Caucasians than in African-Americans (77). In the same subjects, brachycephaly was found to be associated with an increased AHI in Caucasians but not in African-Americans (78).

Similarly, a cross-sectional study of New Zealand Maori (Polynesian) and European (Caucasian) men showed that small reductions in mandibular prognathism and a wider bony nasal aperture represent major factors associated with OSAS in Polynesian men, whereas in the Caucasian group OSAS was associated with a larger neck circumference and a reduced retropalatal airway size (79). In addition, BMI was a stronger predictor of OSAS severity in Caucasian men compared with that in Polynesian men (79).

Ethnicity is associated with hypertension prevalence and is an important independent contributor to OSAS prevalence (47,80–82). The prevalence of OSAS in Japanese hypertensive patients was around 10% (83), which was one-third that of the Western hypertensive participants of the New York Sleep Heart Health Study (84). However, in the largest cross-sectional study involving a total of 6132 participants in China, the prevalence of hypertension in patients with OSAS was 56.2% (85), which is comparable to Western countries. The discrepancies among these results may need to be confirmed in other trials with larger number of patients.

## Various categories of hypertension in OSAS

In normal subjects, blood pressure decreases during sleep by 10%–20% of the awake value and increases promptly on waking. An absence of this nocturnal dip in blood pressure correlates directly with the amount of deep sleep and inversely with indices of sleep fragmentation (86). Thus, there is great interest in the clinical observation and pathophysiological mechanisms underlying dipping and non-dipping patterns of ambulatory blood pressure profiles. In OSAS patients, various categories of hypertension were characterized.

### Nocturnal hypertension

A nighttime fall of blood pressure (dipping) is normal. On the contrary, reduced nocturnal BP (non-dipping) or even higher nocturnal BP than daytime BP is an undoubted risk factor for hypertensive patients due to the end-organ damage and subsequent cardiovascular events (87–89). A blunted nocturnal BP dipping phenomenon is common in hypertensive patients (90). The differences between non-dipping and dipping may be related to the following: 1) alterations in the autonomic nervous function, 2) congestive heart failure, 3) chronic kidney disease, and 4) OSAS. Strikingly, the nocturnal BP profile described in studies of 24-hour blood pressure measurements in OSAS patients is similar to that in non-dipping hypertensive patients.

In the Wisconsin Sleep Cohort Study, Young and colleagues (28) found a dose-response relationship between sleep-disordered breathing and 24-hour blood pressure, independent of known confounding factors. Baguet et al. found that 42% of apneic patients had clinical hypertension (37). In the study, 42% of the OSAS patients showed office hypertension, while 58% of subjects had daytime hypertension, and 76% of patients had nighttime hypertension.

Also, diastolic, systolic, and mean blood pressure values during sleep were significantly related to apnea-hypopnea index and age (91). Another study revealed that blood pressure night/day ratios in patients were associated with severity of OSAS (92). Moreover, a casual relationship between the increasing respiratory disturbance index and the average 24 hour systolic blood pressure was only observed in non-dipping individuals and hypertensive OSAS subjects (93). The repeated end-apneic arousal and/or hypoxic asphyxia and the subsequent sleep fragmentation contributed to nocturnal and diurnal elevation of BP (94). Furthermore, the hypertension is associated with a greater exacerbation of short-term variability during sleep in OSAS patients (39).

Moreover, the relationship between OSAS and nocturnal hypertension might differ between males and females. Interestingly, Portaluppi et al. studied 100 new cases of hypertension in men and found that non-dipping individuals exhibited a blunting of nocturnal blood pressure dipping phenomenon and an increased variability in BP associated with OSAS (29). This suggested that hypertensive non-dipping individuals had a high probability of coexisting sleep-disordered breathing. However, a significant relationship of nighttime/daytime blood pressure difference and AHI existed in men but not in women (95).

**Figure 2.**
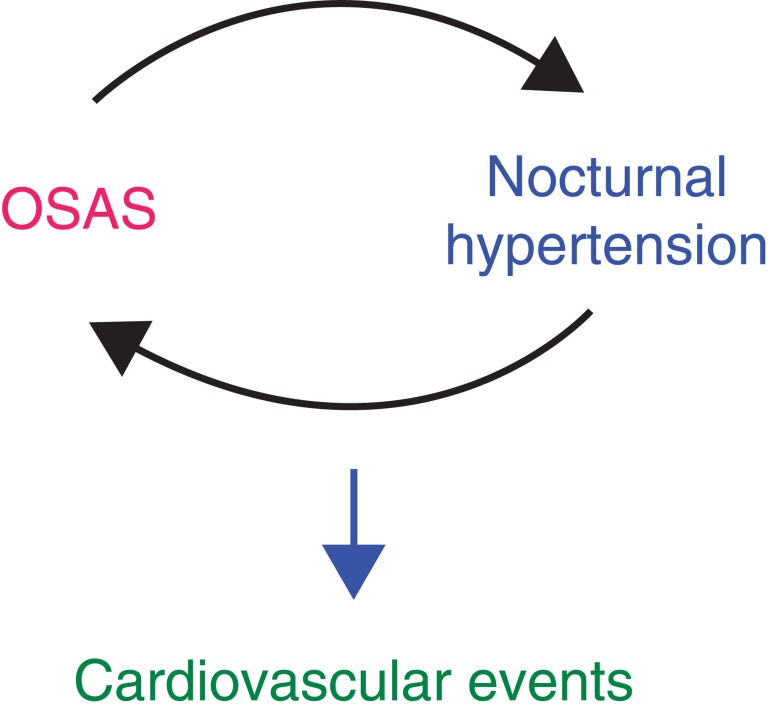
A schematic link between OSAS and nocturnal hypertension leading to cardiovascular events.

OSAS-related hypoxemia and hypercapnia, sleep fragmentation, increased sympathetic activity, chemoreflex activation, and nighttime blood pressure surges may be involved in the increased peripheral vascular tone (nocturnal hypertension) and subsequent cardiovascular events in OSAS patients (Figure 2). Cardiovascular events of relevance include increased incidence of platelet activation, obstructive cardiomyopathy, cardiac arrhythmias, myocardial ischemia or/and infarction, and sudden cardiac death (4,17,96–99). OSAS may also increase the incidence of hemorrhagic and ischemic stroke (100–102) and subsequent neurological injury and cognitive impairment (103,104).

### Resistant hypertension

OSAS is an important secondary cause of resistant hypertension and is particularly common in patients with resistant hypertension (105). Resistant hypertension, also called refractory hypertension, occurs where high blood pressure is not returned to within normal ranges with use of three drugs, including diuretics. A significant gender difference is typical, with OSAS being more prevalent and more severe in male than in female patients (106). Two cross-sectional studies demonstrate that the more severe the OSAS, the less likely blood pressure could be controlled despite increasing the number of antihypertensive agents (107,108). OSAS was extremely common in subjects with resistant hypertension, and a significant correlation between plasma aldosterone concentration and OSAS severity was observed in subjects with resistant hypertension but not in control subjects (109), suggesting that aldosterone excess may contribute to OSAS severity.

### Masked hypertension

Masked hypertension is defined as a normal clinic blood pressure and elevated out-of-clinic blood pressure, assessed using self-monitoring of blood pressure by the patients at home (110,111). Masked hypertension includes stress-induced hypertension, morning hypertension, and nocturnal hypertension, which is defined as a sleeping blood pressure >120/70 mmHg. Recently, Baguet et al. showed a large incidence of masked hypertension, as determined by ambulatory BP monitoring (ABPM), in patients with OSAS who were considered normotensive. The authors concluded that masked hypertension is frequently underestimated in OSAS and is nearly always present when clinic blood pressure is >125/83 mmHg (112).

A recent study revealed that among apparently normotensive male OSAS patients, masked hypertension is present in one-third of patients and there is a progressive impairment of arterial stiffness in OSAS patients with masked hypertension, indicating that the diagnosis of masked hypertension may be underestimated in OSAS patients and that OSAS has an association with arterial stiffness independent of masked hypertension (113). Moreover, obesity may be involved in the relationship between OSAS and masked hypertension. Although common etiologies of masked hypertension include stressful living situations, sleep apnea resulting from obesity is also a possible etiology (114). However, the relationship between masked hypertension and OSAS needs more study.

### Pulmonary hypertension

The first report regarding OSAS and pulmonary hypertension was in 1988. No significant correlation existed between pulmonary arterial pressure and the apnea index (115). However, while intravascular pulmonary arterial pressure decreased during apneas and increased at the resumption of breathing, transmural pulmonary arterial pressure showed a progressive increase during apneas that decreased once ventilation had been resumed, suggesting pulmonary arterial pressure might be related with OSAS (116). The pulmonary artery hypertension was linked to the presence of an obstructive ventilatory pattern, hypoxemia, and hypercapnia, while the severity of OSAS plays only a minor role (117). In a study that recruited 92 consecutive patients, OSAS was found to be an important independent risk factor for pulmonary hypertension (118).

## Mechanism underlying the link between OSAS and hypertension

The main acute physiological consequences of OSAS are intermittent hypoxia, intrapleural pressure changes, and arousals, which might induce endothelial dysfunction, sympathetic activation, lipid metabolism dysfunction, increased oxidative stress, etc. (Figure 1). All of these consequences could increase the artery tone and arterial stiffness, and thereby increase the risk of systemic hypertension and further cardiovascular diseases such as stroke and atherosclerosis.

### Intermittent hypoxia

Intermittent hypoxia, particularly if associated with episodes of intermittent reoxygenation, is one of the most important features in OSAS (119). Intermittent hypoxia causes remarkable blood oxygen desaturation, commonly within a cycle time of less than 1 min. To a certain degree, the intermittent hypoxia induced a repetitive hypoxia/reoxygenation cycle in OSAS, resembling ischemia-reperfusion injury, by promoting the production of reactive oxygen species (ROS), activating systemic inflammation, and ultimately impairing endothelial function. Apart from the induction of systemic inflammation and oxidative stress, intermittent hypoxia increased the plasma level of vasoconstrictive endothelin-1 (120). Additionally, intermittent hypoxia has been proposed to enhance peripheral chemoreceptors and the sympathetic nervous system activity (121). Moreover, hypoxia-induced cellular responses, such as apoptosis (122) and autophagy (123,124), have to be considered.

### Endothelial dysfunction

Endothelial dysfunction is a systemic pathological state of the vascular endothelium and can be broadly defined as an imbalance between vasorelaxation and vasoconstriction substances produced by the endothelium (125). Endothelial dysfunction is an early marker of vascular damage that precedes clinically overt vascular disease and might be an important promoter of cardiovascular events in patients with OSAS (4,126). The strong association between OSAS and cardiovascular diseases may be due to significant endothelial dysfunction in OSAS patients and therefore is accumulating evidence for the association of OSAS and the impaired endothelial function/reduced endothelial repair capacity.

A previous study showed that even mild OSAS was associated with reduced endothelium-dependent vasodilatation (127). Greater impairment of endothelial function was undoubtedly associated with more severe OSAS (128–137). Studies on hypertensive patients with OSAS revealed that there indeed is an association between OSAS and endothelial dysfunction. Furthermore, in normotensive patients with OSAS, endothelium-dependent vasodilatation, measured by forearm blood flow, was impaired (138,139).

Nitric oxide (NO), the most important vasodilatory molecule synthesized by the endothelium, decreased in patients with OSAS (138,140,141). In addition, the level and activity of endothelial progenitor cells, a population of rare cells that circulate in the blood with the ability to differentiate into endothelial cells, decreased in patients with OSAS (134,142–144). Many above-mentioned signaling pathway proteins, including tumor necrosis factor, interleukins IL-1, IL-6, and IL-8, nuclear factor κB (NF-κB), the renin–angiotensin system, and hypoxia-inducible factor-1 (HIF-1) participate in the molecular mechanisms underlying the OSAS-induced endothelial dysfunction (4,145). These signaling proteins will be discussed in the next section.

### Inflammation and oxidative stress

The evidence that OSAS is associated with increased oxidative stress and inflammation is based on findings from both animals and humans. Promoting inflammation and oxidative stress is a major cause of endothelial dysfunction by decreasing NO availability and reducing endothelial vasodilatation capacity. Cells exposed to intermittent hypoxia demonstrated selective activation of the pro-inflammatory transcription factor NF-κB, whereas the adaptive regulator HIF-1 was not activated (146). In addition, elevated NF-κB activity induced by chronic intermittent hypoxia was accompanied by increased expression of inducible nitric oxide synthase (iNOS) level, a putative and important NF-κB-dependent inflammatory and oxidative protein (147).

Moreover, NF-κB could further stimulate the production of pro-inflammatory mediators such as IL-8 and intercellular adhesion molecule 1 (ICAM-1) (4). TNF-alpha levels are independently associated with excessive daytime sleepiness, and IL-8 has shown elevated levels in patients with OSAS compared with controls (148). A recent study raised the possibility that the obese OSAS patients might have elevated TNF-alpha levels compared to BMI-matched controls (149). These factors may contribute to the hypertension in OSAS. C-reactive protein (CRP) plays an important role in hypertension (149,150) and is correlated with OSAS. Shamsuzzaman et al. reported that plasma CRP levels were significantly higher in patients with OSAS than in controls (150), suggesting that the severity of OSAS is proportional to the CRP level, which was confirmed later by numerous cross-sectional, case-control, and non-randomized interventional studies (151-155). Apart from these systemic investigations, cellular studies on lung might also provide some association between inflammation and OSAS (156,157).

## Management and treatment of OSAS

### Non-drug therapy

Weight loss is an effective treatment of obesity in OSAS patients. Weight loss improves significantly sleep apnea and has favorable effects on blood pressure and baroreflex sensitivity (158). The improvement of obstructive sleep apnea after weight loss might be related to improvement in pharyngeal and glottic function (159). Avoidance of alcohol is to be recommended as deterioration of OSAS occurs by alcohol abuse (160,161).

### Continuous positive airway pressure (CPAP) therapy

The most common and highly efficient therapeutic procedure of eliminating airway obstruction is continuous positive airway pressure (CPAP) therapy, a form of treatment first described in 1981 by Sullivan et al. (162). This therapy exerts a blood pressure-lowering effect, reduces nocturnal sympathetic nerve traffic, blunts blood pressure surges, decreases nocturnal blood pressure surge, and improves cardiovascular prognosis in many OSAS patients (163–165). The benefit was larger in patients with more severe sleep apnea than in those who had less severe apnea, but was independent of the baseline blood pressure (166).

However, there are also some studies reporting negative effects (167,168). Lack of concordance may be due to heterogeneity of selected subjects and study design. Thus, three meta-analyses were employed to summarize findings of intervention studies in CPAP treatment. Bazzano et al. reported that mean net change in systolic blood pressure for those treated with CPAP application of 2–24 weeks (818 patients) compared with controls was –2.46 mmHg (*P* < 0.05) (169). Another meta-analysis included 12 trials (572 patients) and reported a significant –1.69 mmHg decrease in mean BP, with similar reductions in SBP and DBP (*P* < 0.05) (170).

On the contrary, Alajmi et al. reported that the effects of CPAP on BP in OSAS patients (572 patients) were modest and not statistically significant (*P* = 0.23) (171). The authors considered that in unselected patients with sleep apnea, CPAP may have modest effects on BP, whereas they could not exclude the possibility that certain subgroups of patients may have more robust responses to CPAP (171). Obviously, although CPAP has been the first-line therapeutic strategy for OSAS, the beneficial effect of CPAP remains an open question.

## Conclusions

Over the last decade, there has been increased interest in OSAS-related research and increased understanding of the OSAS-related cardiovascular complications. Because OSAS is associated with hypertension and hypertension associated end-stage organ diseases such as stroke, coronary heart disease, and arrhythmia, the employment of CPAP is highly encouraged as CPAP therapy seems to assist blood pressure control at least in those with severe apnea, resistant hypertension, and daytime sleepiness. This field continues to be updated monthly. A number of key questions, however, remains to be resolved. More clinical research is warranted to characterize more fully the underlying mechanisms and to develop practicable strategies for OSAS treatment.

## References

[1] Parati G, Lombardi C, Narkiewicz K (2007). Sleep apnea: epidemiology, pathophysiology, and relation to cardiovascular risk. Am J Physiol Regul Integr Comp Physiol.

[2] Weiss JW, Liu MD, Huang J (2007). Physiological basis for a causal relationship of obstructive sleep apnoea to hypertension. Exp Physiol.

[3] Lanfranchi P, Somers VK (2001). Obstructive sleep apnea and vascular disease. Respir Res.

[4] Kohler M, Stradling JR (2010). Mechanisms of vascular damage in obstructive sleep apnea. Nat Rev Cardiol.

[5] Lam JC, Sharma SK, Lam B (2010). Obstructive sleep apnoea: definitions, epidemiology & natural history. Indian J Med Res.

[6] (1999). Sleep-related breathing disorders in adults: recommendations for syndrome definition and measurement techniques in clinical research. The Report of an American Academy of Sleep Medicine Task Force. Sleep.

[7] Baguet JP, Narkiewicz K, Mallion JM (2006). Update on hypertension management: obstructive sleep apnea and hypertension. J Hypertens.

[8] Young T, Palta M, Dempsey J, Skatrud J, Weber S, Badr S (1993). The occurrence of sleep-disordered breathing among middle-aged adults. N Engl J Med.

[9] Ip MS, Lam B, Lauder IJ, Tsang KW, Chung KF, Mok YW (2001). A community study of sleep-disordered breathing in middle-aged Chinese men in Hong Kong. Chest.

[10] Bearpark H, Elliott L, Grunstein R, Cullen S, Schneider H, Althaus W (1995). Snoring and sleep apnea. A population study in Australian men. Am J Respir Crit Care Med.

[11] Bixler EO, Vgontzas AN, Ten Have T, Tyson K, Kales A (1998). Effects of age on sleep apnea in men: I. Prevalence and severity. Am J Respir Crit Care Med.

[12] Ohayon MM, Guilleminault C, Priest RG, Caulet M (1997). Snoring and breathing pauses during sleep: telephone interview survey of a United Kingdom population sample. BMJ.

[13] Ip MS, Lam B, Tang LC, Lauder IJ, Ip TY, Lam WK (2004). A community study of sleep-disordered breathing in middle-aged Chinese women in Hong Kong: prevalence and gender differences. Chest.

[14] Udwadia ZF, Doshi AV, Lonkar SG, Singh CI (2004). Prevalence of sleep-disordered breathing and sleep apnea in middle-aged urban Indian men. Am J Respir Crit Care Med.

[15] Kim J, In K, You S, Kang K, Shim J, Lee S (2004). Prevalence of sleep-disordered breathing in middle-aged Korean men and women. Am J Respir Crit Care Med.

[16] Punjabi NM (2008). The epidemiology of adult obstructive sleep apnea. Proc Am Thorac Soc.

[17] Somers VK, White DP, Amin R, Abraham WT, Costa F, Culebras A (2008). Sleep apnea and cardiovascular disease: an American Heart Association/American College Of Cardiology Foundation Scientific Statement from the American Heart Association Council for High Blood Pressure Research Professional Education Committee, Council on Clinical Cardiology, Stroke Council, and Council On Cardiovascular Nursing. In collaboration with the National Heart, Lung, and Blood Institute National Center on Sleep Disorders Research (National Institutes of Health). Circulation.

[18] Brooks D, Horner RL, Kozar LF, Render-Teixeira CL, Phillipson EA (1997). Obstructive sleep apnea as a cause of systemic hypertension. Evidence from a canine model. J Clin Invest.

[19] Carley DW, Trbovic SM, Radulovacki M (1996). Hydralazine reduces elevated sleep apnea index in spontaneously hypertensive (SHR) rats to equivalence with normotensive Wistar-Kyoto rats. Sleep.

[20] Troncoso Brindeiro CM, da Silva AQ, Allahdadi KJ, Youngblood V, Kanagy NL (2007). Reactive oxygen species contribute to sleep apnea-induced hypertension in rats. Am J Physiol Heart Circ Physiol.

[21] Silverberg DS, Oksenberg A, Iaina A (1998). Sleep-related breathing disorders as a major cause of essential hypertension: fact or fiction?. Curr Opin Nephrol Hypertens.

[22] Fletcher EC, DeBehnke RD, Lovoi MS, Gorin AB (1985). Undiagnosed sleep apnea in patients with essential hypertension. Ann Intern Med.

[23] Kales A, Bixler EO, Cadieux RJ, Schneck DW, Shaw LC, Locke TW (1984). Sleep apnoea in a hypertensive population. Lancet.

[24] Lavie P, Ben-Yosef R, Rubin AE (1984). Prevalence of sleep apnea syndrome among patients with essential hypertension. Am Heart J.

[25] Williams AJ, Houston D, Finberg S, Lam C, Kinney JL, Santiago S (1985). Sleep apnea syndrome and essential hypertension. Am J Cardiol.

[26] Peppard PE, Young T, Palta M, Skatrud J (2000). Prospective study of the association between sleep-disordered breathing and hypertension. N Engl J Med.

[27] Hla KM, Young TB, Bidwell T, Palta M, Skatrud JB, Dempsey J (1994). Sleep apnea and hypertension. A population-based study. Ann Intern Med.

[28] Young T, Peppard P, Palta M, Hla KM, Finn L, Morgan B (1997). Population-based study of sleep-disordered breathing as a risk factor for hypertension. Arch Intern Med.

[29] Portaluppi F, Provini F, Cortelli P, Plazzi G, Bertozzi N, Manfredini R (1997). Undiagnosed sleep-disordered breathing among male nondippers with essential hypertension. J Hypertens.

[30] Hedner J, Bengtsson-Bostrom K, Peker Y, Grote L, Rastam L, Lindblad U (2006). Hypertension prevalence in obstructive sleep apnoea and sex: a population-based case-control study. Eur Respir J.

[31] Nieto FJ, Herrington DM, Redline S, Benjamin EJ, Robbins JA (2004). Sleep apnea and markers of vascular endothelial function in a large community sample of older adults. Am J Respir Crit Care Med.

[32] Tanigawa T, Tachibana N, Yamagishi K, Muraki I, Kudo M, Ohira T (2004). Relationship between sleep-disordered breathing and blood pressure levels in community-based samples of Japanese men. Hypertens Res.

[33] Gottlieb DJ, Redline S, Nieto FJ, Baldwin CM, Newman AB, Resnick HE (2006). Association of usual sleep duration with hypertension: the Sleep Heart Health Study. Sleep.

[34] Kamil MA, Teng CL, Hassan SA (2007). Snoring and breathing pauses during sleep in the Malaysian population. Respirology.

[35] Cui R, Tanigawa T, Sakurai S, Yamagishi K, Imano H, Ohira T (2008). Associations of sleep-disordered breathing with excessive daytime sleepiness and blood pressure in Japanese women. Hypertens Res.

[36] Sharabi Y, Scope A, Chorney N, Grotto I, Dagan Y (2003). Diastolic blood pressure is the first to rise in association with early subclinical obstructive sleep apnea: lessons from periodic examination screening. Am J Hypertens.

[37] Baguet JP, Hammer L, Levy P, Pierre H, Rossini E, Mouret S (2005). Night-time and diastolic hypertension are common and underestimated conditions in newly diagnosed apnoeic patients. J Hypertens.

[38] Sin DD, Fitzgerald F, Parker JD, Newton GE, Logan AG, Floras JS (2003). Relationship of systolic BP to obstructive sleep apnea in patients with heart failure. Chest.

[39] Planes C, Leroy M, Fayet G, Aegerter P, Foucher A, Raffestin B (2002). Exacerbation of sleep-apnoea related nocturnal blood-pressure fluctuations in hypertensive subjects. Eur Respir J.

[40] Bonsignore MR, Parati G, Insalaco G, Marrone O, Castiglioni P, Romano S (2002). Continuous positive airway pressure treatment improves baroreflex control of heart rate during sleep in severe obstructive sleep apnea syndrome. Am J Respir Crit Care Med.

[41] Eckert DJ, Malhotra A (2008). Pathophysiology of adult obstructive sleep apnea. Proc Am Thorac Soc.

[42] Bixler EO, Vgontzas AN, Lin HM, Ten Have T, Rein J, Vela-Bueno A (2001). Prevalence of sleep-disordered breathing in women: effects of gender. Am J Respir Crit Care Med.

[43] McLaren SM, McPherson FM, Sinclair F, Ballinger BR (1981). Prevalence and severity of incontinence among hospitalized, female psychogeriatric patients. Health Bull (Edinb).

[44] Peppard PE, Young T, Palta M, Dempsey J, Skatrud J (2000). Longitudinal study of moderate weight change and sleep-disordered breathing. JAMA.

[45] Ancoli-Israel S, DuHamel ER, Stepnowsky C, Engler R, Cohen-Zion M, Marler M (2003). The relationship between congestive heart failure, sleep apnea, and mortality in older men. Chest.

[46] Eikermann M, Jordan AS, Chamberlin NL, Gautam S, Wellman A, Lo YL (2007). The influence of aging on pharyngeal collapsibility during sleep. Chest.

[47] Hajjar I, Kotchen TA (2003). Trends in prevalence, awareness, treatment, and control of hypertension in the United States, 1988–2000. JAMA.

[48] Wang Y, Beydoun MA (2007). The obesity epidemic in the United States—gender, age, socioeconomic, racial/ethnic, and geographic characteristics: a systematic review and meta-regression analysis. Epidemiol Rev.

[49] Mokdad AH, Bowman BA, Ford ES, Vinicor F, Marks JS, Koplan JP (2001). The continuing epidemics of obesity and diabetes in the United States. JAMA.

[50] Ferretti A, Giampiccolo P, Cavalli A, Milic-Emili J, Tantucci C (2001). Expiratory flow limitation and orthopnea in massively obese subjects. Chest.

[51] Strohl KP, Redline S (1996). Recognition of obstructive sleep apnea. Am J Respir Crit Care Med.

[52] Tishler PV, Larkin EK, Schluchter MD, Redline S (2003). Incidence of sleep-disordered breathing in an urban adult population: the relative importance of risk factors in the development of sleep-disordered breathing. JAMA.

[53] Young T, Peppard PE, Taheri S (2005). Excess weight and sleep-disordered breathing. J Appl Physiol.

[54] Schwab RJ, Gupta KB, Gefter WB, Metzger LJ, Hoffman EA, Pack AI (1995). Upper airway and soft tissue anatomy in normal subjects and patients with sleep-disordered breathing. Significance of the lateral pharyngeal walls. Am J Respir Crit Care Med.

[55] Schwab RJ, Gefter WB, Hoffman EA, Gupta KB, Pack AI (1993). Dynamic upper airway imaging during awake respiration in normal subjects and patients with sleep disordered breathing. Am Rev Respir Dis.

[56] Sharp JT, Henry JP, Sweany SK, Meadows WR, Pietras RJ (1964). Effects of mass loading the respiratory system in man. J Appl Physiol.

[57] Thut DC, Schwartz AR, Roach D, Wise RA, Permutt S, Smith PL (1993). Tracheal and neck position influence upper airway airflow dynamics by altering airway length. J Appl Physiol.

[58] Series F, Marc I (1994). Influence of lung volume dependence of upper airway resistance during continuous negative airway pressure. J Appl Physiol.

[59] Series F, Cormier Y, Desmeules M (1990). Influence of passive changes of lung volume on upper airways. J Appl Physiol.

[60] Yaggi HK, Strohl KP (2010). Adult obstructive sleep apnea/hypopnea syndrome: definitions, risk factors, and pathogenesis. Clin Chest Med.

[61] Mortimore IL, Marshall I, Wraith PK, Sellar RJ, Douglas NJ (1998). Neck and total body fat deposition in nonobese and obese patients with sleep apnea compared with that in control subjects. Am J Respir Crit Care Med.

[62] Flemons WW, Whitelaw WA, Brant R, Remmers JE (1994). Likelihood ratios for a sleep apnea clinical prediction rule. Am J Respir Crit Care Med.

[63] Schwab RJ (1996). Properties of tissues surrounding the upper airway. Sleep.

[64] Schafer H, Pauleit D, Sudhop T, Gouni-Berthold I, Ewig S, Berthold HK (2002). Body fat distribution, serum leptin, and cardiovascular risk factors in men with obstructive sleep apnea. Chest.

[65] (2007). Asia Pacific Cohort Studies Collaboration. The burden of overweight and obesity in the Asia-Pacific region. Obes Rev.

[66] Lam JC, Lam B, Lam CL, Fong D, Wang JK, Tse HF (2006). Obstructive sleep apnea and the metabolic syndrome in community-based Chinese adults in Hong Kong. Respir Med.

[67] Yue W, Liu H, Zhang J, Zhang X, Wang X, Liu T (2008). Association study of serotonin transporter gene polymorphisms with obstructive sleep apnea syndrome in Chinese Han population. Sleep.

[68] Sharma SK, Kumpawat S, Banga A, Goel A (2006). Prevalence and risk factors of obstructive sleep apnea syndrome in a population of Delhi, India. Chest.

[69] Bhushan B, Misra A, Guleria R (2010). Obstructive sleep apnea is independently associated with the metabolic syndrome in obese Asian Indians in northern India. Metab Syndr Relat Disord.

[70] Bhushan B, Guleria R, Misra A, Pandey RM, Luthra K, Vikram NK (2009). Obstructive sleep apnoea correlates with C-reactive protein in obese Asian Indians. Nutr Metab Cardiovasc Dis.

[71] Bhushan B, Guleria R, Misra A, Luthra K, Vikram NK (2009). TNF-alpha gene polymorphism and TNF-alpha levels in obese Asian Indians with obstructive sleep apnea. Respir Med.

[72] Reddy EV, Kadhiravan T, Mishra HK, Sreenivas V, Handa KK, Sinha S (2009). Prevalence and risk factors of obstructive sleep apnea among middle-aged urban Indians: a community-based study. Sleep Med.

[73] Sharma SK, Mishra HK, Sharma H, Goel A, Sreenivas V, Gulati V (2008). Obesity, and not obstructive sleep apnea, is responsible for increased serum hs-CRP levels in patients with sleep-disordered breathing in Delhi. Sleep Med.

[74] Kim SH, Cho GY, Baik I, Kim J, Kim SJ, Lee JB (2010). Association of coronary artery calcification with obstructive sleep apnea and obesity in middle-aged men. Nutr Metab Cardiovasc Dis.

[75] Chung S, Yoon IY, Lee CH, Kim JW (2010). The association of nocturnal hypoxemia with arterial stiffness and endothelial dysfunction in male patients with obstructive sleep apnea syndrome. Respiration.

[76] Sakakibara H, Tong M, Matsushita K, Hirata M, Konishi Y, Suetsugu S (1999). Cephalometric abnormalities in non-obese and obese patients with obstructive sleep apnoea. Eur Respir J.

[77] Redline S, Tishler PV, Hans MG, Tosteson TD, Strohl KP, Spry K (1997). Racial differences in sleep-disordered breathing in African-Americans and Caucasians. Am J Respir Crit Care Med.

[78] Cakirer B, Hans MG, Graham G, Aylor J, Tishler PV, Redline S (2001). The relationship between craniofacial morphology and obstructive sleep apnea in whites and in African-Americans. Am J Respir Crit Care Med.

[79] Coltman R, Taylor DR, Whyte K, Harkness M (2000). Craniofacial form and obstructive sleep apnea in Polynesian and Caucasian men. Sleep.

[80] (1977). Race, education and prevalence of hypertension. Am J Epidemiol.

[81] Bassett DR, Fitzhugh EC, Crespo CJ, King GA, McLaughlin JE (2002). Physical activity and ethnic differences in hypertension prevalence in the United States. Prev Med.

[82] Kramer H, Han C, Post W, Goff D, Diez-Roux A, Cooper R (2004). Racial/ethnic differences in hypertension and hypertension treatment and control in the multi-ethnic study of atherosclerosis (MESA). Am J Hypertens.

[83] Kario K, Ishikawa J, Hoshide S, Ishikawa S, Eguchi K, Pickering TG Vascular consequences of sleep disordered breathing—Sleep apnea syndrome and hypertensive target organ damage in Japan.

[84] Kario K, Rapoport D, Schwartz JE Sleep-disordered breathing as a determinant of nondipping status of nocturnal blood pressure independent of age and body mass index: The New York Sleep Heart Health Study (SHHS).

[85] Chen BY, He QY (2007). A multi-center study on the association between sleep apnea and prevalence of hypertension. Chinese Journal of Tuberculosis and Respiratory Diseases.

[86] Loredo JS, Nelesen R, Ancoli-Israel S, Dimsdale JE (2004). Sleep quality and blood pressure dipping in normal adults. Sleep.

[87] Kario K, Matsuo T, Kobayashi H, Imiya M, Matsuo M, Shimada K (1996). Nocturnal fall of blood pressure and silent cerebrovascular damage in elderly hypertensive patients. Advanced silent cerebrovascular damage in extreme dippers. Hypertension.

[88] Boggia J, Li Y, Thijs L, Hansen TW, Kikuya M, Bjorklund-Bodegard K (2007). Prognostic accuracy of day versus night ambulatory blood pressure: a cohort study. Lancet.

[89] Nagai M, Hoshide S, Ishikawa J, Shimada K, Kario K (2008). Ambulatory blood pressure as an independent determinant of brain atrophy and cognitive function in elderly hypertension. J Hypertens.

[90] de la Sierra A, Redon J, Banegas JR, Segura J, Parati G, Gorostidi M (2009). Prevalence and factors associated with circadian blood pressure patterns in hypertensive patients. Hypertension.

[91] Lavie P, Yoffe N, Berger I, Peled R (1993). The relationship between the severity of sleep apnea syndrome and 24-h blood pressure values in patients with obstructive sleep apnea. Chest.

[92] Pankow W, Nabe B, Lies A, Becker H, Kohler U, Kohl FV (1997). Influence of sleep apnea on 24-hour blood pressure. Chest.

[93] Suzuki M, Guilleminault C, Otsuka K, Shiomi T (1996). Blood pressure “dipping” and “non-dipping” in obstructive sleep apnea syndrome patients. Sleep.

[94] Noda A, Yasuma F, Okada T, Yokota M (2000). Influence of movement arousal on circadian rhythm of blood pressure in obstructive sleep apnea syndrome. J Hypertens.

[95] Sforza E, Lugaresi E (1995). Determinants of the awakening rise in systemic blood pressure in obstructive sleep apnea syndrome. Blood Press.

[96] Chan HS, Chiu HF, Tse LK, Woo KS (1991). Obstructive sleep apnea presenting with nocturnal angina, heart failure, and near-miss sudden death. Chest.

[97] Peled N, Abinader EG, Pillar G, Sharif D, Lavie P (1999). Nocturnal ischemic events in patients with obstructive sleep apnea syndrome and ischemic heart disease: effects of continuous positive air pressure treatment. J Am Coll Cardiol.

[98] Steiner S, Schueller PO, Hennersdorf MG, Behrendt D, Strauer BE (2008). Impact of obstructive sleep apnea on the occurrence of restenosis after elective percutaneous coronary intervention in ischemic heart disease. Respir Res.

[99] Szuhay GP (2009). Idiopathic intracranial hypertension without papilledema and sleep apnea in children: a case series of a single observer. Ann Neurol.

[100] Neau JP, Paquereau J, Meurice JC, Chavagnat JJ, Gil R (2002). Stroke and sleep apnoea: cause or consequence?. Sleep Med Rev.

[101] Netzer NC (2010). Impaired nocturnal cerebral hemodynamics during long obstructive apneas: the key to understanding stroke in OSAS patients?. Sleep.

[102] Wierzbicka A, Rola R, Wichniak A, Richter P, Ryglewicz D, Jernajczyk W (2006). The incidence of sleep apnea in patients with stroke or transient ischemic attack. J Physiol Pharmacol.

[103] Gagnon JF, Vendette M, Postuma RB, Desjardins C, Massicotte-Marquez J, Panisset M (2009). Mild cognitive impairment in rapid eye movement sleep behavior disorder and Parkinson's disease. Ann Neurol.

[104] Hutchison KN, Avison M, Cannistraci C (2009). Neural correlates of adaptation to sleep deprivation in obstructive sleep apnea—a pilot study. Ann Neurol.

[105] Calhoun DA, Jones D, Textor S, Goff DC, Murphy TP, Toto RD (2008). Resistant hypertension: diagnosis, evaluation, and treatment: a scientific statement from the American Heart Association Professional Education Committee of the Council for High Blood Pressure Research. Circulation.

[106] Logan AG, Perlikowski SM, Mente A, Tisler A, Tkacova R, Niroumand M (2001). High prevalence of unrecognized sleep apnoea in drug-resistant hypertension. J Hypertens.

[107] Grote L, Hedner J, Peter JH (2000). Sleep-related breathing disorder is an independent risk factor for uncontrolled hypertension. J Hypertens.

[108] Lavie P, Hoffstein V (2001). Sleep apnea syndrome: a possible contributing factor to resistant. Sleep.

[109] Pratt-Ubunama MN, Nishizaka MK, Boedefeld RL, Cofield SS, Harding SM, Calhoun DA (2007). Plasma aldosterone is related to severity of obstructive sleep apnea in subjects with resistant hypertension. Chest.

[110] Pickering TG, Miller NH, Ogedegbe G, Krakoff LR, Artinian NT, Goff D (2008). Call to action on use and reimbursement for home blood pressure monitoring: a joint scientific statement from the American Heart Association, American Society Of Hypertension, and Preventive Cardiovascular Nurses Association. Hypertension.

[111] Pickering TG, Eguchi K, Kario K (2007). Masked hypertension: a review. Hypertens Res.

[112] Baguet JP, Levy P, Barone-Rochette G, Tamisier R, Pierre H, Peeters M (2008). Masked hypertension in obstructive sleep apnea syndrome. J Hypertens.

[113] Drager LF, Diegues-Silva L, Diniz PM, Bortolotto LA, Pedrosa RP, Couto RB (2010). Obstructive sleep apnea, masked hypertension, and arterial stiffness in men. Am J Hypertens.

[114] Mak RH, Bakris G (2010). Pediatrics: masked hypertension: a risk factor in children with CKD. Nat Rev Nephrol.

[115] Weitzenblum E, Krieger J, Apprill M, Vallee E, Ehrhart M, Ratomaharo J (1988). Daytime pulmonary hypertension in patients with obstructive sleep apnea syndrome. Am Rev Respir Dis.

[116] Marrone O, Bellia V, Ferrara G, Milone F, Romano L, Salvaggio A (1989). Transmural pressure measurements. Importance in the assessment of pulmonary hypertension in obstructive sleep apneas. Chest.

[117] Chaouat A, Weitzenblum E, Krieger J, Oswald M, Kessler R (1996). Pulmonary hemodynamics in the obstructive sleep apnea syndrome. Results in 220 consecutive patients. Chest.

[118] Sanner BM, Doberauer C, Konermann M, Sturm A, Zidek W (1997). Pulmonary hypertension in patients with obstructive sleep apnea syndrome. Arch Intern Med.

[119] Garvey JF, Taylor CT, McNicholas WT (2009). Cardiovascular disease in obstructive sleep apnoea syndrome: the role of intermittent hypoxia and inflammation. Eur Respir J.

[120] Kanagy NL, Walker BR, Nelin LD (2001). Role of endothelin in intermittent hypoxia-induced hypertension. Hypertension.

[121] Lesske J, Fletcher EC, Bao G, Unger T (1997). Hypertension caused by chronic intermittent hypoxia—influence of chemoreceptors and sympathetic nervous system. J Hypertens.

[122] Gozal D, Row BW, Kheirandish L, Liu R, Guo SZ, Qiang F (2003). Increased susceptibility to intermittent hypoxia in aging rats: changes in proteasomal activity, neuronal apoptosis and spatial function. J Neurochem.

[123] Ng S, Wu YT, Chen B, Zhou J, Shen HM (2011). Impaired autophagy due to constitutive mTOR activation sensitizes TSC2-null cells to cell death under stress. Autophagy.

[124] Lee SJ, Kim HP, Jin Y, Choi AM, Ryter SW (2011). Beclin 1 deficiency is associated with increased hypoxia-induced angiogenesis. Autophagy.

[125] Deanfield J, Donald A, Ferri C, Giannattasio C, Halcox J, Halligan S (2005). Endothelial function and dysfunction. Part I: Methodological issues for assessment in the different vascular beds: a statement by the Working Group on Endothelin and Endothelial Factors of the European Society of Hypertension. J Hypertens.

[126] Budhiraja R, Parthasarathy S, Quan SF (2007). Endothelial dysfunction in obstructive sleep apnea. J Clin Sleep Med.

[127] Duchna HW, Stoohs R, Guilleminault C, Christine Anspach M, Schultze-Werninghaus G, Orth M (2006). Vascular endothelial dysfunction in patients with mild obstructive sleep apnea syndrome. Wien Med Wochenschr.

[128] Ip MS, Tse HF, Lam B, Tsang KW, Lam WK (2004). Endothelial function in obstructive sleep apnea and response to treatment. Am J Respir Crit Care Med.

[129] Zhang XL, Yin KS, Mao H, Wang H, Yang Y (2004). Effect of continuous positive airway pressure treatment on vascular endothelial function in patients with obstructive sleep apnea hypopnea syndrome and coronary artery disease. Chin Med J (Engl).

[130] Duchna HW, Orth M, Schultze-Werninghaus G, Guilleminault C, Stoohs RA (2005). Long-term effects of nasal continuous positive airway pressure on vasodilatory endothelial function in obstructive sleep apnea syndrome. Sleep Breath.

[131] Itzhaki S, Lavie L, Pillar G, Tal G, Lavie P (2005). Endothelial dysfunction in obstructive sleep apnea measured by peripheral arterial tone response in the finger to reactive hyperemia. Sleep.

[132] Grebe M, Eisele HJ, Weissmann N, Schaefer C, Tillmanns H, Seeger W (2006). Antioxidant vitamin C improves endothelial function in obstructive sleep apnea. Am J Respir Crit Care Med.

[133] Itzhaki S, Dorchin H, Clark G, Lavie L, Lavie P, Pillar G (2007). The effects of 1-year treatment with a herbst mandibular advancement splint on obstructive sleep apnea, oxidative stress, and endothelial function. Chest.

[134] Jelic S, Padeletti M, Kawut SM, Higgins C, Canfield SM, Onat D (2008). Inflammation, oxidative stress, and repair capacity of the vascular endothelium in obstructive sleep apnea. Circulation.

[135] Kohler M, Craig S, Nicoll D, Leeson P, Davies RJ, Stradling JR (2008). Endothelial function and arterial stiffness in minimally symptomatic obstructive sleep apnea. Am J Respir Crit Care Med.

[136] Trzepizur W, Gagnadoux F, Abraham P, Rousseau P, Meslier N, Saumet JL (2009). Microvascular endothelial function in obstructive sleep apnea: impact of continuous positive airway pressure and mandibular advancement. Sleep Med.

[137] Priou P, Gagnadoux F, Tesse A, Mastronardi ML, Agouni A, Meslier N (2010). Endothelial dysfunction and circulating microparticles from patients with obstructive sleep apnea. Am J Pathol.

[138] Carlson JT, Rangemark C, Hedner JA (1996). Attenuated endothelium-dependent vascular relaxation in patients with sleep apnoea. J Hypertens.

[139] Kato M, Roberts-Thomson P, Phillips BG, Haynes WG, Winnicki M, Accurso V (2000). Impairment of endothelium-dependent vasodilation of resistance vessels in patients with obstructive sleep apnea. Circulation.

[140] Teramoto S, Kume H, Matsuse T, Ishii T, Miyashita A, Akishita M (2003). Oxygen administration improves the serum level of nitric oxide metabolites in patients with obstructive sleep apnea syndrome. Sleep Med.

[141] Ohike Y, Kozaki K, Iijima K, Eto M, Kojima T, Ohga E (2005). Amelioration of vascular endothelial dysfunction in obstructive sleep apnea syndrome by nasal continuous positive airway pressure—possible involvement of nitric oxide and asymmetric NG, NG-dimethylarginine. Circ J.

[142] de la Pena M, Barcelo A, Barbe F, Pierola J, Pons J, Rimbau E (2008). Endothelial function and circulating endothelial progenitor cells in patients with sleep apnea syndrome. Respiration.

[143] Martin K, Stanchina M, Kouttab N, Harrington EO, Rounds S (2008). Circulating endothelial cells and endothelial progenitor cells in obstructive sleep apnea. Lung.

[144] Kim JH, Yu YS, Mun JY, Kim KW (2011). Autophagy-induced regression of hyaloid vessels in early ocular development. Autophagy.

[145] Baguet JP, Barone-Rochette G, Pepin JL (2009). Hypertension and obstructive sleep apnoea syndrome: current perspectives. J Hum Hypertens.

[146] Ryan S, Taylor CT, McNicholas WT (2005). Selective activation of inflammatory pathways by intermittent hypoxia in obstructive sleep apnea syndrome. Circulation.

[147] Greenberg H, Ye X, Wilson D, Htoo AK, Hendersen T, Liu SF (2006). Chronic intermittent hypoxia activates nuclear factor-kappaB in cardiovascular tissues in vivo. Biochem Biophys Res Commun.

[148] Ryan S, Taylor CT, McNicholas WT (2006). Predictors of elevated nuclear factor-kappaB-dependent genes in obstructive sleep apnea syndrome. Am J Respir Crit Care Med.

[149] Steiropoulos P, Papanas N, Nena E, Antoniadou M, Serasli E, Papoti S (2010). Inflammatory markers in middle-aged obese subjects: does obstructive sleep apnea syndrome play a role?. Mediators Inflamm.

[150] Shamsuzzaman AS, Winnicki M, Lanfranchi P, Wolk R, Kara T, Accurso V (2002). Elevated C-reactive protein in patients with obstructive sleep apnea. Circulation.

[151] Teramoto S, Yamamoto H, Ouchi Y (2003). Increased C-reactive protein and increased plasma interleukin-6 may synergistically affect the progression of coronary atherosclerosis in obstructive sleep apnea syndrome. Circulation.

[152] Yokoe T, Minoguchi K, Matsuo H, Oda N, Minoguchi H, Yoshino G (2003). Elevated levels of C-reactive protein and interleukin-6 in patients with obstructive sleep apnea syndrome are decreased by nasal continuous positive airway pressure. Circulation.

[153] Kokturk O, Ciftci TU, Mollarecep E, Ciftci B (2005). Elevated C-reactive protein levels and increased cardiovascular risk in patients with obstructive sleep apnea syndrome. Int Heart J.

[154] Punjabi NM, Beamer BA (2007). C-reactive protein is associated with sleep disordered breathing independent of adiposity. Sleep.

[155] Chung S, Yoon IY, Shin YK, Lee CH, Kim JW, Lee T (2007). Endothelial dysfunction and C-reactive protein in relation with the severity of obstructive sleep apnea syndrome. Sleep.

[156] Fortuna AM, Miralda R, Calaf N, Gonzalez M, Casan P, Mayos M (2011). Airway and alveolar nitric oxide measurements in obstructive sleep apnea syndrome. Respir Med.

[157] Luciani A, Villella VR, Esposito S, Brunetti-Pierri N, Medina DL, Settembre C (2011). Cystic fibrosis: a disorder with defective autophagy. Autophagy.

[158] Kansanen M, Vanninen E, Tuunainen A, Pesonen P, Tuononen V, Hartikainen J (1998). The effect of a very low-calorie diet-induced weight loss on the severity of obstructive sleep apnoea and autonomic nervous function in obese patients with obstructive sleep apnoea syndrome. Clin Physiol.

[159] Rubinstein I, Colapinto N, Rotstein LE, Brown IG, Hoffstein V (1988). Improvement in upper airway function after weight loss in patients with obstructive sleep apnea. Am Rev Respir Dis.

[160] Teschler H, Berthon-Jones M, Wessendorf T, Meyer HJ, Konietzko N (1996). Influence of moderate alcohol consumption on obstructive sleep apnoea with and without AutoSet nasal CPAP therapy. Eur Respir J.

[161] Nerfeldt P, Graf P, Borg S, Friberg D (2004). Prevalence of high alcohol and benzodiazepine consumption in sleep apnea patients studied with blood and urine tests. Acta Otolaryngol.

[162] Sullivan CE, Issa FG, Berthon-Jones M, Eves L (1981). Reversal of obstructive sleep apnoea by continuous positive airway pressure applied through the nares. Lancet.

[163] Wilcox I, Grunstein RR, Hedner JA, Doyle J, Collins FL, Fletcher PJ (1993). Effect of nasal continuous positive airway pressure during sleep on 24-hour blood pressure in obstructive sleep apnea. Sleep.

[164] Faccenda JF, Mackay TW, Boon NA, Douglas NJ (2001). Randomized placebo-controlled trial of continuous positive airway pressure on blood pressure in the sleep apnea-hypopnea syndrome. Am J Respir Crit Care Med.

[165] Becker HF, Jerrentrup A, Ploch T, Grote L, Penzel T, Sullivan CE (2003). Effect of nasal continuous positive airway pressure treatment on blood pressure in patients with obstructive sleep apnea. Circulation.

[166] Pepperell JC, Ramdassingh-Dow S, Crosthwaite N, Mullins R, Jenkinson C, Stradling JR (2002). Ambulatory blood pressure after therapeutic and subtherapeutic nasal continuous positive airway pressure for obstructive sleep apnoea: a randomised parallel trial. Lancet.

[167] Robinson GV, Smith DM, Langford BA, Davies RJ, Stradling JR (2006). Continuous positive airway pressure does not reduce blood pressure in nonsleepy hypertensive OSA patients. Eur Respir J.

[168] Campos-Rodriguez F, Grilo-Reina A, Perez-Ronchel J, Merino-Sanchez M, Gonzalez-Benitez MA, Beltran-Robles M (2006). Effect of continuous positive airway pressure on ambulatory BP in patients with sleep apnea and hypertension: a placebo-controlled trial. Chest.

[169] Bazzano LA, Khan Z, Reynolds K, He J (2007). Effect of nocturnal nasal continuous positive airway pressure on blood pressure in obstructive sleep apnea. Hypertension.

[170] Haentjens P, Van Meerhaeghe A, Moscariello A, De Weerdt S, Poppe K, Dupont A (2007). The impact of continuous positive airway pressure on blood pressure in patients with obstructive sleep apnea syndrome: evidence from a meta-analysis of placebo-controlled randomized trials. Arch Intern Med.

[171] Alajmi M, Mulgrew AT, Fox J, Davidson W, Schulzer M, Mak E (2007). Impact of continuous positive airway pressure therapy on blood pressure in patients with obstructive sleep apnea hypopnea: a meta-analysis of randomized controlled trials. Lung.

